# Trends in Glycemia between 2002 and 2016 among Incident Youth Cohorts Early in the Course of Type 1 Diabetes: The SEARCH for Diabetes in Youth Study

**DOI:** 10.1155/2022/8554991

**Published:** 2022-07-22

**Authors:** Daria Igudesman, Beth A. Reboussin, Katherine J. Souris, Catherine Pihoker, Lawrence Dolan, Jean M. Lawrence, Sharon Saydah, Dana Dabelea, Santica Marcovina, Noémie Clouet-Foraison, Faisal S. Malik, Elizabeth J. Mayer-Davis

**Affiliations:** ^1^Department of Nutrition, University of North Carolina at Chapel Hill, Chapel Hill, NC 27599, USA; ^2^Department of Biostatistics and Data Science, Wake Forest School of Medicine, Winston-Salem, NC 27101, USA; ^3^Department of Pediatrics, University of Washington, Seattle Children's, Seattle, WA 98105, USA; ^4^Division of Endocrinology, Department of Pediatrics, University of Cincinnati College of Medicine, Cincinnati, OH 45229, USA; ^5^Department of Research & Evaluation, Kaiser Permanente Southern California, Pasadena CA 91101, USA; ^6^Division of Diabetes Translation, National Center for Chronic Disease Prevention and Health Promotion, Centers for Disease Control and Prevention, Atlanta, GA 30333, USA; ^7^Department of Epidemiology, Colorado School of Public Health, University of Colorado, Aurora, CO 80045, USA; ^8^Division of Metabolism, Endocrinology and Nutrition, University of Washington, Seattle, WA 98195, USA; ^9^Department of Medicine, University of North Carolina at Chapel Hill, Chapel Hill, NC 27599, USA

## Abstract

**Objective:**

Hyperglycemia early in the course of type 1 diabetes (T1D) may increase the risk of cardiometabolic complications later in life. We tested the hypothesis that there were temporal trends in population-level glycemia and insulin pump use near T1D diagnosis among incident youth cohorts diagnosed between 2002 and 2016.

**Methods:**

Weighted and adjusted regression models were applied to data from the SEARCH for Diabetes in Youth study to analyze trends in hemoglobin A1c (HbA1c), suboptimal glycemia (HbA1c > 9% or not), and insulin pump use among youth with T1D within 30 months of diagnosis. We tested the interaction of year with race and ethnicity, sex, and insulin regimen to assess potential disparities.

**Results:**

Among the 3,956 youth with T1D, there was a small, clinically insignificant reduction in HbA1c between 2002 (7.9% ± 1.5) and 2016 (7.8% ± 2.4) (fully adjusted change by year (-0.013% [95% CI -0.026, -0.0008], *p* = 0.04). The proportion of youth with suboptimal glycemia increased with each year, but the adjusted odds did not change. Insulin pump use increased more than fivefold. Although interaction effects of time with race and ethnicity, sex, and insulin regimen were not detected, in 2016, suboptimal glycemia was 4.3 and 1.8 times more prevalent among Black and Hispanic than among non-Hispanic White youth, respectively.

**Conclusions:**

There was not a clinically significant population-level improvement in glycemia across incident youth cohorts early in the course of T1D, despite severalfold increases in insulin pump use. Comprehensive clinical interventions to improve glycemia early in the T1D course and address disparities are urgently needed.

## 1. Introduction

Among youth with type 1 diabetes (T1D), increased HbA1c is associated with acute and chronic complications [1]. However, fewer than 25% of youth with T1D meet the American Diabetes Association's HbA1c target of <7.0% (53 mmol/mol) [2]. An even smaller proportion of minority youth meet glycemic recommendations—a rising concern given that the incidence of T1D is increasing at faster rates in racial and ethnic minority groups compared to non-Hispanic White youth [3–6].

Glycemia has not improved among youth with longstanding T1D despite advancements in diabetes technologies [7]—nearly one-third to one-half have an HbA1c ≥ 9.5% (80 mmol/mol) [5, 8]. Glycemia proximal to T1D diagnosis is also critical to manage given that a metabolic memory may persist beyond the period of initial diagnosis to influence future risk of diabetes-related complications [9]. However, no studies have assessed population-level glycemia trends early in the course of T1D. Therefore, the present study was aimed at evaluating whether HbA1c and the prevalence of suboptimal glycemia (HbA1c > 9%) changed between 2002 and 2016 across incident cohorts of SEARCH youth within 30 months of T1D diagnosis and at examining potential disparities by race and ethnicity, sex, and insulin regimen.

## 2. Methods

### 2.1. Study Population

Detailed SEARCH study methods are published elsewhere [10]. Briefly, SEARCH is a population-based registry characterizing the incidence and prevalence of youth-onset diabetes diagnosed at <20 years of age, incorporating a prospective cohort study. Youth with type 1 or type 2 diabetes were identified at five US sites (Ohio, Colorado, Washington, South Carolina, and California). This analysis focuses on youth diagnosed with T1D in 2002-2006, 2008, 2012, and 2016 and participated in a baseline research visit within 30 months of their diabetes diagnosis. HbA1c was measured in a central laboratory (Northwest Lipid Metabolism and Diabetes Research Laboratories, University of Washington, Seattle, Washington).

Per study protocol, all registered youth were invited to participate in a visit if they were diagnosed in 2002-2006 or 2008; all registered minority youth, non-Hispanic White youth older than age 10, and a random 50% sample of non-Hispanic White youth aged 10 or younger were invited to participate in 2012; and all registered minority youth and a random one-third of non-Hispanic White youth were invited to participate in 2016. Non-Hispanic White youth in 2012 and 2016, and younger non-Hispanic White youth in 2012 only, were purposefully undersampled in these years because sufficient numbers of youth with these characteristics had been recruited to power the main SEARCH study analyses. Youth were included in the present analysis only once, in the year in which they were diagnosed. Local institutional review board approval was obtained for each center. Written informed consent was obtained from participants aged ≥18 years. Assent with parental written informed consent was obtained for participants < 18 years.

### 2.2. Insulin Regimen

Insulin regimen was derived from participant report at the baseline visit and dichotomized as insulin pump or other regimens.

### 2.3. Statistical Analyses

Glycemia was analyzed as continuous HbA1c (%) or dichotomous suboptimal glycemia (HbA1c > 9.0% or not). This definition of suboptimal glycemia has been used in prior studies [11, 12].

Analyses were weighted to account for the undersampling of non-Hispanic White youth in 2012 and 2016 and of younger non-Hispanic White youth in 2012 using survey sampling procedures in SAS 9.4 (SAS Institute, Cary, NC). Time trends were examined using linear regression for continuous HbA1c and logistic regression for suboptimal glycemia and insulin pump use, regressing these measures on index (i.e., diagnosis) year). We additionally used linear regression to assess whether diabetes duration changed over time, given that HbA1c covaries positively with diabetes duration, particularly within and shortly after the first year of diagnosis, when there is residual beta cell function [13]. Index year was treated as continuous to examine whether trends were linear and account for uneven spacing between years.

To account for residual beta cell function in the year following T1D diagnosis (i.e., the honeymoon period) [13], we first adjusted models by diabetes duration at the study visit (Model 1). Model 2 was further adjusted for demographic variables (age, sex, race and ethnicity, and clinic site). Race and ethnicity were categorized as Black, Hispanic, Other or Unknown, or non-Hispanic White. Given sample size limitations and the small numbers of youth who identified with an Asian or Pacific Islander (*n* = 93) or Native American (*n* = 19) race, these two categories were collapsed into Other and Unknown race and ethnicity. Given our interest in disparities, we examined interactions between index year and race and ethnicity, sex, and insulin regimen on the outcomes of HbA1c and suboptimal glycemia in fully adjusted models. An *α* < 0.05was considered to be statistically significant for both main and interaction effects.

## 3. Results

### 3.1. Participant Characteristics

Among the 3,956 youth with T1D included in the analysis, diabetes duration increased across the incident youth cohorts (Table 1). There was a fivefold increase in the proportion of youth using insulin pumps between 2002 (6.3%) and 2016 (34.5%) (*p* = 0.01). Use of insulin pump therapy increased among all racial and ethnic subgroups (total *n* = 3,850 due to missing data for insulin regimen). In 2016, a smaller proportion of Black youth (16.7%), Hispanic youth (26.9%), and youth with an Other or Unknown race and ethnicity (29.4%) reported using an insulin pump compared to non-Hispanic White youth (40.0%).

### 3.2. HbA1c

Mean HbA1c did not improve across incident years (7.9% ± 1.5 [63 mmol/mol ± 16.4] in 2002 and 7.8% ± 2.4 [62 mmol/mol ± 26.2] in 2016, *p* = 0.6 for trend). Model 1 adjusted for diabetes duration also did not show a statistically significant change in HbA1c over time (data not shown). Model 2 further adjusted for potential demographic confounders showed a statistically significant but small reduction in HbA1c with increasing index year (-0.013% [95% CI -0.026, -0.0008], *p* = 0.04).

### 3.3. Suboptimal Glycemia

There was a statistically significant increase over time in the unadjusted proportion of SEARCH youth with T1D who had suboptimal glycemia (14.1% in 2002 and 18.1% in 2016, *p* = 0.03 for trend). Model 1 adjusted for diabetes duration and Model 2 further adjusted for potential demographic confounders did not show a statistically significant change in the prevalence of suboptimal glycemia over time (fully adjusted odds ratio 0.99 [95% CI 0.97, 1.02], *p* = 0.58). Given that diabetes duration increased over time, we removed this covariate from Model 2 in a sensitivity analysis to assess whether diabetes duration was an important explanatory variable in the change in suboptimal glycemia. Results were not different with and without diabetes duration in the model, which demonstrated that the attenuation of the adjusted Model 2 to nonsignificance was not due to changes in diabetes duration over time.

### 3.4. Subgroup Analyses by Race and Ethnicity, Sex, and Insulin Regimen

**Figure 1:**
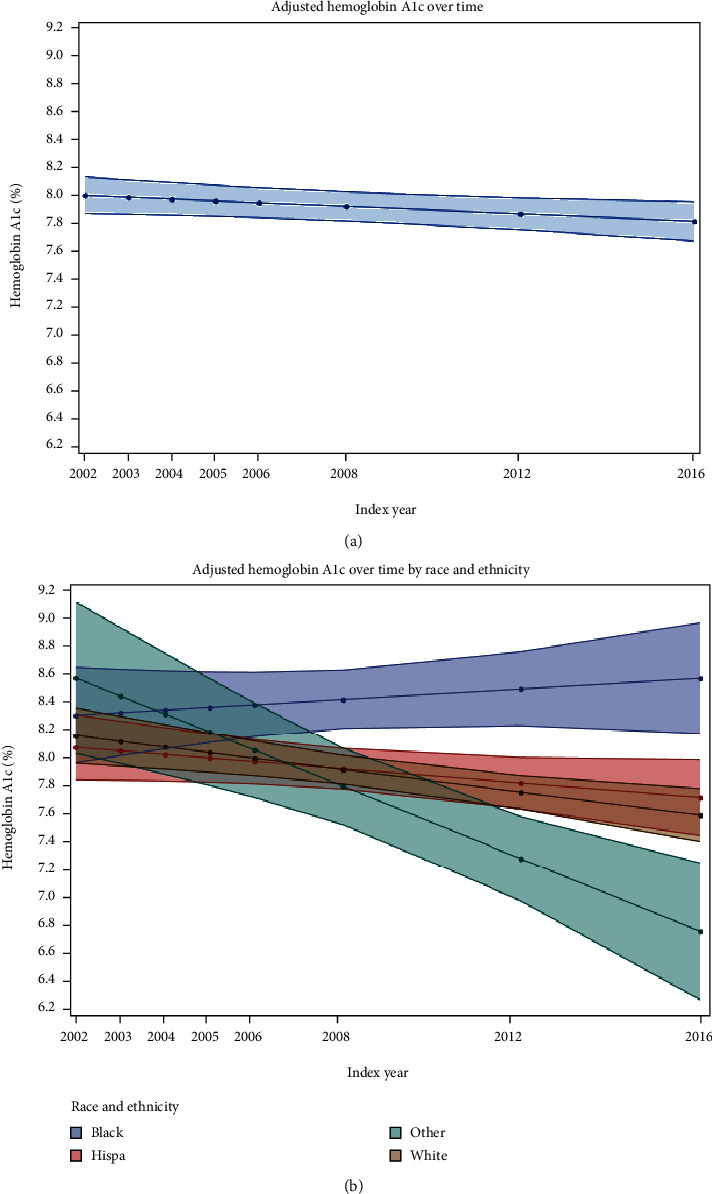
Mean hemoglobin A1c across incident youth cohorts with type 1 diabetes, overall and by race and ethnicity subgroup. (a) There was a small but clinically nonsignificant reduction in the fully adjusted overall change in hemoglobin A1c (HbA1c) over time. (b) The interaction of time with race and ethnicity for HbA1c was not statistically significant, but racial and ethnic disparities persisted over time. In particular, mean HbA1c was higher among Black youth than among non-Hispanic White youth in all index years. The Other race and ethnicity subgroup includes youth with an Other, Unknown, Asian or Pacific Islander, or Native American race and a Hispanic ethnicity. Sample sizes for subgroups across index years ranged from *n* = 40-93 for Black youth, *n* = 42-117 for Hispanic youth, *n* = 7-30 for youth with an Other race, and *n* = 149-475 for non-Hispanic White youth. Abbreviations: Hispa—Hispanic; Other—Other or Unknown; White—non-Hispanic.

Two-way interaction effects of year with race and ethnicity, sex, and binary insulin regimen were not statistically significant in crude or adjusted interaction models (all *p* > 0.1). HbA1c and the crude proportion of youth with suboptimal glycemia decreased with time among youth in the Other or Unknown race and ethnicity subgroup: HbA1c was 9.7% (95% CI 7.9, 11.5) in 2002 and 7.2% (95% CI 6.4, 8.0) in 2016 in this subgroup (*p* = 0.002 for trend); suboptimal glycemia was 38.5% in 2002 and 11.1% in 2016, *p* = 0.002 for trend) and did not change in any other racial or ethnic subgroup. Mean HbA1c and the proportion of youth in suboptimal glycemia were numerically higher among Black youth in all index years and among Hispanic youth in all index years except for 2004, compared to non-Hispanic White youth (Figure 1).

## 4. Discussion

Glycemia did not improve meaningfully between 2002 and 2016 across incident youth cohorts assessed shortly after diagnosis, a time that encompasses the period of partial remission (i.e., the “honeymoon period”). This is worrisome given that a metabolic memory may cause hyperglycemia near T1D diagnosis to confer increased cardiometabolic risk later in life [6]. Given the residual insulin production that characterizes the honeymoon period, HbA1c often remains near-normal during this time and the need for exogenous insulin is greatly reduced [13]; however, mean HbA1c remained nearly one percentage point higher than recommended across most index years despite the fact that all youth were within 30 months of T1D diagnosis. Although insulin pump use increased in all racial and ethnic subgroups, disparities in glycemia and insulin pump use persisted over time.

This study includes several limitations and strengths. We lacked continuous glucose monitoring (CGM) data, which may better represent comprehensive glycemic management than HbA1c by elucidating daily metrics such as time in target glucose range and glycemic variability [14]. We assume that CGM use increased over time in our study sample concurrently with increased insulin pump use [7, 14]. We lacked a sufficiently large sample size to stratify by all original racial and ethnic subgroups. The small numbers of youth with an Other or Unknown race may have precluded detection of a statistically significant interaction effect of year with race and ethnicity for the glycemic outcomes. Although diabetes duration increased with time—an indication that the duration of time between T1D diagnosis and measurement of HbA1c at the SEARCH baseline visit increased—we adjusted for this variable in statistical models, which did not change results. It was important to assess change in diabetes duration over time as part of our analysis given that HbA1c levels tends to be lower earlier in the T1D course due to residual beta cell function, which declines over time [13]. Strengths of this study include the oversampling of underrepresented groups and its population-based nature, which allows for generalizability of our findings to the broader population of US youth early in the T1D course.

Our results are consistent with the concept that technology use alone does not necessarily improve glycemic outcomes. This finding is echoed by a recent study which found that *in silico* assignment of SEARCH participants with T1D who were members of racial and ethnic minority subgroups to the insulin regimen (i.e., to more frequent insulin pump use) of non-Hispanic White participants did not fully reduce disparities in HbA1c (it did so by one-third) [15]. Likewise, disparities in glycemic outcomes among minority youth with T1D are not fully explained by biological [16], clinical, or demographic factors [17]. Adding a focus on alleviating health disparities to systematic quality improvement efforts in multidisciplinary care processes [18, 19] may help to improve HbA1c at a population level near the time of T1D diagnosis, thereby improving quality of life and reducing future microvascular and cardiometabolic risk for all.

## Figures and Tables

**Table 1:** Distribution of clinical and demographic characteristics between 2002 and 2016 among incident youth cohorts with type 1 diabetes.

	2002	2003	2004	2005	2006	2008	2012	2016	*p* value
All (*N*)	446	472	468	453	484	664	592	377	—
Diabetes duration (months): mean (SD)	13.0 (0.33)	8.9 (0.28)	8.3 (0.26)	8.2 (0.25)	10.3 (0.24)	8.6 (0.22)	10.6 (0.32)	13.5 (0.50)	<0.0001
Using insulin pump: *N* (%)									
Overall	27 (6.3)	52 (11.3)	36 (7.8)	40 (9.0)	71 (15.0)	90 (13.9)	119 (22.6)	105 (34.5)	<0.0001
Black	1 (2.6)	1 (2.2)	0 (0.0)	1 (2.1)	1 (2.3)	4 (6.4)	8 (9.1)	14 (16.7)	<0.0001
Hispanic	0 (0.0)	1 (2.2)	2 (4.0)	4 (7.4)	5 (8.1)	4 (3.9)	12 (13.8)	28 (26.9)	<0.0001
Other or Unknown	0 (0.0)	0 (0.0)	0 (0.0)	0 (0.0)	0 (0.0)	6 (40.0)	4 (13.3)	5 (29.4)	0.0002
White	26 (7.7)	50 (14.0)	34 (9.9)	35 (10.4)	65 (18.5)	76 (16.3)	95 (27.2)	58 (40.0)	<0.0001
HbA1c: mean (95% CI)									
Overall	7.9 (7.7, 8.0)	7.7 (7.6, 7.8)	7.7 (7.6, 7.9)	7.6 (7.4, 7.7)	8.0 (7.9, 8.2)	7.7 (7.5, 7.8)	7.8 (7.7, 8.0)	7.8 (7.6, 8.0)	0.60
Black	8.7 (8.0, 9.5)	8.1 (7.5, 8.7)	8.2 (7.6, 8.9)	8.1 (7.6, 8.7)	9.2 (8.3, 10.0)	8.4 (7.9, 9.0)	8.3 (7.8, 8.7)	9.0 (8.4, 9.5)	0.21
Hispanic	8.1 (7.6, 8.6)	8.0 (7.5, 8.6)	7.6 (7.2, 8.1)	8.1 (7.7, 8.5)	8.2 (7.8, 8.6)	7.7 (7.4, 8.0)	8.0 (7.6, 8.4)	7.9 (7.6, 8.2)	0.74
Other or Unknown	9.7 (7.9, 11.5)	8.6 (7.4, 9.7)	8.5 (7.8, 9.2)	7.3 (6.0, 8.7)	7.5 (6.8, 8.2)	7.8 (7.2, 8.4)	7.4 (6.9, 7.9)	7.2 (6.4, 8.0)	0.002
White	7.7 (7.5, 7.8)	7.6 (7.4, 7.7)	7.6 (7.5, 7.8)	7.4 (7.3, 7.6)	7.9 (7.7, 8.1)	7.5 (7.4, 7.6)	7.7 (7.6, 7.9)	7.6 (7.3, 7.8)	0.95
Suboptimal glycemia > 9%: *N* (%)									
Overall	63 (14.1)	66 (14.0)	69 (14.7)	66 (14.6)	105 (21.7)	112 (16.9)	120 (19.3)	88 (18.1)	0.03
Black	16 (40.0)	12 (25.5)	18 (32.7)	15 (31.3)	22 (50.0)	26 (39.4)	27 (30.3)	45 (48.4)	0.10
Hispanic	11 (26.2)	14 (29.8)	8 (16.0)	16 (28.6)	18 (28.1)	19 (17.8)	25 (27.8)	24 (20.5)	0.49
Other or Unknown	5 (38.5)	4 (30.8)	7 (43.8)	1 (14.3)	1 (5.9)	2 (12.5)	4 (13.3)	2 (11.1)	0.03
White	31 (8.8)	36 (9.9)	36 (10.4)	34 (9.9)	64 (17.8)	65 (13.7)	64 (16.2)	17 (11.4)	0.12

Abbreviation: HbA1c: hemoglobin A1c; ADA: American Diabetes Association. Data were obtained from *N* = 3,956 SEARCH participants with type 1 diabetes who had a baseline visit and a hemoglobin A1c measurement within 30 months of diagnosis. Results were computed using weighted survey procedures. For categorical variables, counts are unweighted, but proportions are weighted. Other race includes Asian/Pacific Islander, Native American, and Other race.

## Data Availability

The data that support the findings of this study are available from the SEARCH for Diabetes in Youth Study but restrictions apply to the availability of these data, which were used under license for the current study, and so are not publicly available. Data are however available from the authors upon reasonable request and with permission of the SEARCH for Diabetes in Youth Study.
